# Malarial Origin of Rheumatism

**Published:** 1896-01

**Authors:** R. L. Patterson

**Affiliations:** Coyville, Kan.


### MALARIAL ORIGIN OF RHEUMATISM.

Now that tubercle bacilli can be detected in the earliest stages of phthisis, the Klebs-Loefler bacillus decides the presence of diphtheria, the malarial germs of Laveran tell us we have intermittent fever. I recently had two cases of inflammatory rheumatism, and in neither case did I anticipate having a rheumatic diathesis to treat, as they both had malarial paroxysms each alternate day for at least ten days previous to the development of any of the clinical symptoms usually present at the outset of a typical case of inflammatory rheumatic fever. It is now in order for some enthusiastic advocate of the germ theory of disease to discover the rheumatic germ in both cases mentioned. The malarial paroxysms were abated in forty-eight hours administration of

℞. Febraline, ℥vi.

Sig. :—Dessert spoonful every three hours.

To insure the complete and prompt absorption of any of the alkaloids of the cinchona bark, they must be in a liquid form, and febraline is a pleasant liquid form.

*Coyville, Kan., Dec. 8, 1895.* R. L. PATTERSON, M. D.

